# Long‐Term Study on the Fluctuation of Self‐Reported Awake Bruxism in a Cohort of Healthy Young Adults

**DOI:** 10.1111/joor.13872

**Published:** 2024-10-09

**Authors:** Anna Colonna, Frank Lobbezoo, Alessandro Bracci, Marco Ferrari, Matteo Val, Daniele Manfredini

**Affiliations:** ^1^ School of Dentistry, Department of Medical Biotechnologies University of Siena Siena Italy; ^2^ Department of Orofacial Pain and Dysfunction, Academic Centre for Dentistry Amsterdam (ACTA) University of Amsterdam and Vrije Universiteit Amsterdam Amsterdam The Netherlands; ^3^ Department of Neurosciences, School of Dentistry University of Padova Padova Italy

**Keywords:** awake bruxism, awake bruxism behaviours, bruxism, ecological momentary assessment, ecological momentary intervention, smartphone

## Abstract

**Background:**

The introduction of a smartphone‐based ecological momentary assessment (EMA) approach has allowed achieving data on the frequency of different awake bruxism (AB) behaviours (i.e., teeth contact, teeth clenching, teeth grinding, and mandible bracing) reported by an individual in the natural environment.

**Study Objectives:**

The fluctuation of AB reports over time has a certain degree of variability that has never been investigated. Therefore, the aim of this investigation was to assess the long‐term fluctuation of AB behaviours in a population of young adults.

**Methods:**

A smartphone application was used to assess a real‐time report on five specific oral conditions related to AB in a sample of 77 young adults, aged 24.0 ± 0.8 years. Data were recorded over three periods of 7 days, with a three‐month interval for a total of 6 months.

**Results:**

The average frequency of the relaxed condition was 72.9%, 78.2%, and 80.8% at the end of the first, second, and third sessions, respectively. On average, teeth contact and mandible bracing were the most frequently reported conditions, with a mean prevalence of 12.9% and 7%, respectively, whilst the frequency of teeth clenching and teeth grinding was less than 3%. The ANOVA test showed an absence of significant differences (*p* < 0.05) between the three recording periods, and the frequency was in general only moderately variable from day‐to‐day (e.g., the coefficient of variation (CV) for the condition “relaxed jaw muscles” was 0.3). No gender differences were detected either.

**Conclusions:**

Findings suggest that in a population of healthy individuals, the frequency of AB behaviours over a six‐month monitoring period is quite constant. This investigation represents a standpoint for future comparisons on the study of natural fluctuations of AB behaviours as well as on AB frequency in populations with risk/associated factors and possible clinical consequences.

## Graphical abstract

The present investigation presented data on the frequency of AB in a large sample of healthy young adults by adopting an EMA approach, which provides a real‐time evaluation of different AB activities reported by an individual in his/her natural environment, over a six‐month period.

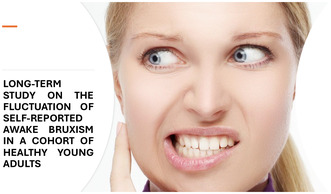

**Abbreviations**
AB: awake bruxism
CI: confidence intervals
CV: coefficient of variation
EMA: ecological momentary assessment
EMI: ecological momentary intervention
SB: sleep bruxism
SD: standard deviation

## 1 Introduction

Bruxism is a much‐debated oral condition that is gaining increasing attention in both research and clinical settings in several medical fields. Recently, some experts were invited to take part in an international consensus meeting. They provided separate definitions for awake bruxism (AB) and sleep bruxism (SB) and also discussed the possible development and refinement of the available assessment approaches [1]. Specifically, AB has been defined as follows:

“Awake bruxism is a masticatory muscle activity during wakefulness that is characterised by repetitive or sustained tooth contact and/or by bracing or thrusting of the mandible and is not a movement disorder in otherwise healthy individuals.”

Bruxism as a jaw‐muscle behaviour in otherwise healthy individuals, which is not necessarily related to pathological and/or clinical consequences, should be measured or evaluated in its continuum and in the natural environment [1, 2, 3, 4, 5, 6]. Under this premise, the updated definition led to an increased focus on the general concepts and assessment strategies of AB [7, 8].

Regarding the assessment strategies, expert consensus suggests that AB can be evaluated with a combination of instrumental (i.e., electromyography) and non‐instrumental (i.e., self‐report and clinical observation) approaches, as well as with ecological momentary assessment (EMA) [1, 2, 4, 7, 8, 9]. Several studies [10, 11, 12, 13, 14, 15, 16, 17, 18] were performed on the use of EMA to assess the frequency of self‐reported oral behaviours that are related to the AB spectrum, since it is a simple method to collect real‐time data in the individual's natural environment. In short, EMA refers to a real‐time report of a behaviour, a sensation, or any condition under study [19]. The principle of EMA is that an individual is requested at fixed or random timepoints, whilst living his/her usual daily routine, to answer questions about what (s)he is currently doing and/or experiencing. As such, multiple recording points during the day, close in time to the experience and in the natural environment, are allowed [18, 20].

In view of this, using EMA strategies helps achieve a better description of AB epidemiology, both at the general population level and in selected groups of individuals with possible clinical consequences and/or potential risk factors for an increase in AB [1, 2, 4].

Recently, EMA strategies focusing on AB were implemented thanks to a smartphone app that sends alerts at random timepoints during the day. Upon alert receipt, the subject has to focus on his/her current condition and tap on the corresponding display icon [10, 11, 12, 13, 14, 15, 16, 17, 18]. Nonetheless, despite the potential advantages of this strategy, only shortterm data have been published so far, whilst studies on the natural fluctuation of AB could benefit a lot from the use of EMA approaches as an instrument to examine the day‐to‐day variability of behaviours over multiple observation periods. The study of natural fluctuation of AB behaviours, as in the case of SB, is fundamental to setting the standard of reference for the assessment (i.e., bruxism status) and management (i.e., evaluating treatment effectiveness) in the clinical setting [3, 21, 22, 23]. It is also interesting to delve deeper into the gender differences as the literature reports contrasting results [10, 13, 17].

Within these premises, this study aimed to evaluate AB behaviours in a large sample of healthy young adults. To pursue this goal, this investigation was designed to assess the fluctuation in the frequency of AB behaviours over three different one‐week sessions at three‐monthly intervals by the adoption of smartphone‐based EMA technology.

## 2 Methods

A sample of healthy young adults who were recruited from amongst the dental students attending the School of Dentistry at the University of Siena, Siena, Italy, underwent three different seven‐day sessions at three‐monthly intervals with a smartphone application (BruxApp, World Medical Applications Srl, Italy) that was specifically developed to report and monitor the frequency of AB behaviours in an individual's natural environment. The research protocol was approved by the Institutional Review Board of the Orofacial Pain Unit, University of Siena, Siena, Italy (IRB protocol code 02‐22). All individuals gave their informed consent in accordance with the Helsinki Declaration and understood that they were free to withdraw from the study at any time. Exclusion criteria were the presence of temporomandibular disorders (TMD) pain, as screened with the Diagnostic Criteria for Temporomandibular Disorders (DC/TMD) guidelines [24], and/or any documented neurological, psychiatric, sleep, or systemic (e.g., rheumatologic, hormonal) diseases.

All subjects received instructions and information on the smartphone application during two dedicated training sessions with the leading investigator (A.C.). In addition, the project coordinator (D.M.) also recorded an educational video to describe how to recognise the five behaviours (https://www.youtube.com/watch?v=xL79AcnpBCY&t=15s). In short, the app is based on the principle of collecting self‐reported experience during everyday life (i.e., “ecological approach”) and sends alerts at random times during the day to alert the individual on the condition of his/her teeth and jaw muscles. The subject must answer by touching the icon on the smartphone display that refers to the current condition of his/her jaw muscles within 5 min from the alert. Answers are related to five oral conditions: relaxed jaw muscles, mandible bracing, teeth contact, teeth clenching, and teeth grinding. The software was programmed to send 20 alerts per day at random intervals to limit expectation bias (e.g., the risk that individuals may modify their behaviours based on the alert expectation, if set at predetermined intervals). Recording time was set from 8.00 to 12.30 and from 14.30 to 22.00. Based on a previous publication on the expected compliance [11], the subjects were requested to give at least 60% of valid answers/day (i.e., the answer must be given within 5 min, otherwise an error message appears on the display). Days with a compliance < 60% were automatically discarded. The app automatically generates one or more additional days until the target of 7 days in which the subjects replied to at least 60% of the total alerts (i.e., a minimum of 12 alerts/day) was reached, in order to complete the seven‐day protocol three times at three‐monthly intervals for a total of 6 months. After the observation period, the software generated an anonymous, preformatted Excel file that participants sent to the leading investigator via a privacy‐protected mail system.

For any additional details on the application, readers are referred to the original publications [9, 25].

### 2.1 Statistical Analysis

The data, obtained from the three different sessions of seven‐day monitoring at three‐monthly intervals, were stored in a MS Excel database (Microsoft Corporation, Redmond, WA, USA). All statistical procedures were performed using the SPSS 25.0 software (IBM, Milan, Italy). A descriptive evaluation of the frequency of each condition (i.e., relaxed jaw muscles, teeth contact, teeth clenching, teeth grinding, and mandible bracing), calculated as a percentage with respect to the answered alerts, was performed in all individuals.

The frequency was calculated for each individual, and individual frequencies were used to calculate an average of the study population on a daily basis for each condition. At the end of the seven‐day collecting period, the mean frequency of each condition was assessed for the study population. Data were reported as mean values of the seven‐day span per condition as previously described by Kaplan and Ohrbach [26] and Bracci et al. [10]. In detail, the mean frequency value is the number of positive responses for each specific behaviour per reporting period.

For each condition, a coefficient of variation (CV; i.e., ratio between SD and mean values over the seven recording days) of frequency data was assessed in order to evaluate the fluctuation within the seven‐day sessions. Gender comparison was performed using the Student's *t*‐test. Analysis of variance (ANOVA) was used to test for significant differences between the three different seven‐day sessions. The level of significance was set at *p* < 0.05.

## 3 Results

Within the 103 students attending the final 3 years of the School of Dentistry, 26 were excluded from the data analysis because of a history of TMD pain (*N* = 9), the presence of systemic rheumatic disease (*N* = 4), or a lack of compliance in replying to the alert sessions (*N* = 13). This led to a final sample of 77 subjects (34 males, 43 females; mean age 24.0 ± 0.8 years) taking part in the study.

The average response rate to the alerts during the three recording sessions was 75.1% (±14.7). In detail, the response rates were 75.5% (±13), 75.8% (±16), and 74.1% (±15) for the first, second, and third sessions, respectively (Table 1).

**TABLE 1:** Mean values of frequency data of positive observations (standard deviations in parenthesis) expressed in percentage for the different awake bruxism (AB) behaviours over the three different seven‐day sessions with a three‐month interval between observation periods (*p* value refers to the ANOVA test). Data refer to a study population‐level average.

Activity	T1	T2	T3	*F*	*p*
Relaxed jaw muscles	72.9 (22.3)	78.2 (21.3)	80.8 (21.2)	2.7	0.070
Teeth contact	15.5 (15.0)	13.1 (16.9)	10.0 (13.9)	2.0	0.136
Teeth clenching	3.6 (10.2)	2.0 (7.1)	2.0 (8.5)	0.9	0.419
Teeth grinding	0.3 (1.5)	0.3 (1.2)	0.2 (1.1)	0.1	0.933
Mandible bracing	7.7 (10.6)	6.4 (11.4)	7.0 (15.3)	0.3	0.764
Frequency of response to alerts	75.5 (13)	75.8 (16)	74.1 (15)		

Abbreviations: T1, first seven‐day monitoring session; T2, second seven‐day monitoring session; T3, third seven‐day monitoring session.

On average, the frequency of the various AB behaviours over the three different seven‐day sessions was as follows: relaxed jaw muscles, 77.3% (±21.7); teeth contact, 12.9% (±15.4); mandible bracing, 7% (±12.6); teeth clenching, 2.5% (±8.7); and teeth grinding, 0.3% (±1.3) (Table 2).

**TABLE 2:** Frequency data expressed in percentage of positive observations (mean values, range, 95% confidence intervals, and coefficient of variation) for the different awake bruxism (AB) behaviours over the three different seven‐day sessions.

Activity	Mean frequency (SD)	Range	95% CI	CV
Relaxed jaw muscles	77.3 (21.7)	0–100	74.5–80.2	0.3
Teeth contact	12.9 (15.4)	0–81	11.1–15.1	1.3
Teeth clenching	2.5 (8.7)	0–70.9	1.4–3.7	3.8
Teeth grinding	0.3 (1.3)	0–12.1	0.1–0.4	3.7
Mandible bracing	7.0 (12.6)	0–76.4	5.6–8.9	1.8
Frequency of response to alerts	75.1 (14.7)	60–100	74.4–75.8	0.2

Abbreviations: CI, confidence intervals; CV, coefficient of variation; SD, standard deviation.

Over the first 7 days (T1), the average frequency of relaxed jaw muscle reports at the study population level was 72.9% (±22.3). Teeth contact (15.5%) and mandible bracing (7.7%) were the most frequent AB behaviours. Three months (T2) and 6 months (T3) later, the frequency of these conditions was as follows: relaxed jaw muscles 78.2% (±21.3) and 80.8% (±21.2), teeth contact 13.1% (±16.9) and 10% (±13.9), and mandible bracing 6.4% (±11.4) and 7% (±15.3), respectively (Table 1).

The CV of the frequency of each condition at the study group level over the three different seven‐day sessions was low for the condition “relaxed jaw muscles” (0.3), whilst it was higher for the behaviours “teeth contact” (1.3), “mandible bracing” (1.8), “teeth grinding” (3.7), and “teeth clenching” (3.8). ANOVA, carried out for the three monitoring sessions (i.e., T1, T2, and T3) showed that there were no statistically significant differences in any of the variables considered. No significant gender differences were detected either.

## 4 Discussion

Although bruxism is an oral condition that is gaining increasing attention in several disciplines, such as dentistry, sleep medicine, neurology, and psychology, there is a paucity of literature data on the epidemiology of AB compared to SB [4, 27]. Data on AB are not easy to summarise due to the adoption of different assessment strategies, mostly based on retrospective self‐report at a single observation point. In view of this, as suggested by several papers, the EMA approach can improve the quantity and quality of data collection, as it provides multiple time point reporting in real time over an observation period [1, 2, 4, 7, 10, 11, 12, 13, 14, 15, 16, 17, 18, 28, 29, 30, 31].

The present investigation assessed the fluctuation of AB behaviours in a sample of healthy young adults using a smartphone‐based application for a real‐time report (i.e., EMA) over an approximate six‐month period. The decision to use the EMA approach over other methods (e.g., electromyographic recordings and a questionnaire at a single observation point) for this specific study was its suitability to be used several times during the six‐month evaluation period. This offers the possibility of evaluating a condition in real time in natural settings for several days, with a potential advantage in terms of ecological validity over other strategies. The fact that data are collected in the everyday (“real world”) environment, as subjects go about their lives, increased the data representativeness to resemble an individual's real life. In addition, it must be pointed out that such an approach allows for the collection of large amounts of data, with thousands of alerts answered with self‐reports of the condition in real time. Based on this, these results are hard to compare with other studies due to the different study designs and the commonly used strategy to collect self‐reported data at single timepoints [32].

Our results suggest that the compliance with the EMA approach has been satisfactory, with an average response rate to the alerts during the three recording sessions of 75.1%, which is in line with what was previously published [11]. Also in line with previous studies [10, 11, 13, 14, 15, 17, 28], the average frequencies of relaxed jaw muscles, as a percentage of answers, were 72.9%, 78.2%, and 80.8% at the end of the first, second, and third sessions, respectively. Concerning the fluctuations over time within each monitoring period, it is interesting to note a very low CV, which means that the frequency of AB behaviours does not change in a clinically relevant way, in line with what was previously suggested [10, 28].

In line with several investigations [10, 13, 14, 15, 17, 28], on average, considering the three sessions, the most frequently reported AB conditions were teeth contact (12.9%) and mandible bracing (7%), whilst the least frequently reported one was teeth grinding (0.3%). The aspect that teeth grinding is rarely reported from different countries in any study populations is a potential clue to discriminate SB from AB in terms of muscle behaviours, etiology, and possible clinical and medical consequences. In addition, as suggested by a recent paper [7], from a dental perspective, this data is important for considering the role of bruxism in relation to tooth wear and implant complications, which are hardly viewed as a consequence of AB.

As far as gender differences are concerned, we did not find any statistically significant differences, in agreement with some previous works [10, 15, 17].

Comparing these data collected in healthy young adults with those gathered in other selected populations, it is noteworthy that the percentage of report of relaxed jaw muscles vs. AB behaviours (i.e., teeth contact, teeth clenching, teeth grinding, and mandible bracing) is reversed when the study sample is represented by patients with myofascial pain and temporomandibular joint (TMJ) pain. Indeed, as suggested by Câmara‐Souza et al. [34], patients with musculoskeletal symptoms have higher AB frequency than individuals without such symptoms (i.e., 62.1% ± 26.8% for TMD patients and 36.2% ± 27.3% for pain‐free subjects), especially characterised by jaw bracing, irrespective of pain location.

Our findings also showed an absence of significant differences between the three recording sessions, but the slight increase in the percentage report of relaxed condition is an interesting aspect to study further in patients' populations. Such data are in line with previously published studies that suggested a potential EMI‐biofeedback effect associated with the prolonged use of EMA approaches [14, 17]. This type of approach in fact allows testing for potential ecological momentary intervention (EMI) biofeedback‐related effects, as experimented in individuals who show potentially damaging behaviours, as a strategy for their identification and change [17, 18, 35]. In this view, this approach may also offer interesting perspectives from a therapeutic viewpoint, which must of course be tested with specific study designs. In particular, it is recommendable that this hypothesis be assessed in selected populations of individuals with high frequency of AB behaviours, also as a strategy to manage possible clinical consequences, such as muscle pain and TMJ problems.

This investigation has a potential shortcoming, represented by the study sample of dental students, which may not be representative of the general population. Nonetheless, findings are quite similar to what has been reported in previous studies at the general population level [33]. Despite the possible improvement that can be achieved in the future by addressing the above limitation, the present investigation is particularly innovative as it evaluated for the first time the fluctuation of AB behaviours (i.e., teeth contact, teeth clenching, teeth grinding, and mandible bracing) in a sample of healthy young adults in such an extended time (i.e., a total of approx. 6 months of monitoring over the three different seven‐day sessions, at a three‐monthly interval).

Observational studies based on large‐scale data collection thanks to the feature of EMA may help setting a reference value for AB frequency to compare future investigations on the epidemiological features of AB. On the other hand, these findings could also be useful for future comparison with selected populations of individuals with a purported higher prevalence of bruxism [30, 34, 36, 37] due to observed consequences (e.g., muscle pain and muscle fatigue), potential risk factors (e.g., psychological issues, smoking habits, dietary), and comorbid conditions (e.g., orofacial pain, sleep disorders, and psychological and social impairment).

## 5 Conclusions

The present paper presented data on the frequency of AB in a sample of healthy young adults by adopting an EMA approach, which provides a real‐time evaluation of different AB activities reported by an individual in his/her natural environment over a six‐month period. Teeth contact and mandible bracing are the most frequently reported AB behaviours, whilst teeth grinding is almost absent during wakefulness. All activities have a non‐significant fluctuation over the three sessions and within the sessions themselves, especially when the “relaxed jaw muscles” condition is concerned.

## Author Contributions

Anna Colonna performed the study and wrote the first draft of the article. Frank Lobbezoo and Marco Ferrari helped conceptualise the investigation and revised the manuscript. Alessandro Bracci conceptualised the investigation and provided software assistance throughout the study. Matteo Val contributed to data collection, recruitment of participants, and manuscript writing. Daniele Manfredini supervised the investigation.

## Ethics Statement

The research protocol was approved by the Institutional Review Board of the Orofacial Pain Unit, University of Siena, Siena, Italy (IRB protocol code 02‐22). All individuals gave their informed consent in accordance with the Helsinki Declaration and understood that they were free to withdraw from the study at any time.

## Conflicts of Interest

Alessandro Bracci took part in the development of the computing utilisation of the Bruxapp software. Anna Colonna, Lobbezoo Frank Lobbezoo, Ferrari, Matteo Val, and Daniele Manfredini do not have any conflict of interest concerning this investigation.

### Peer Review

The peer review history for this article is available at https://www.webofscience.com/api/gateway/wos/peer‐review/10.1111/joor.13872.

## Data Availability

The data that support the findings of this study are available from the corresponding author upon reasonable request.
